# Supplementary material for: Automatic Variable Selection Algorithms in Prognostic Factor Research in Neck Pain
Source: J Clin Med. 2023 Sep 27;12(19):6232. doi: 10.3390/jcm12196232 (PMC10573798; doi:10.3390/jcm12196232)
Supplement: Supplementary file 1 [file jcm-12-06232-s001.zip › jcm-2607027-supplementary.pdf]

Supplementary Material

Automatic variable selection algorithms in prognostic factor research in neck pain

Bernard X.W. Liew[1]*, Francisco M. Kovacs[2], David Rügamer[3], Ana Royuela,[4]

***Correspondence:** Bernard Liew, School of Sport, Rehabilitation and Exercise Sciences, University of Essex, Colchester, Essex, United Kingdom; E-mail: bl19622@essex.ac.uk; liew_xwb@hotmail.com. Tel: +44 120 687 3522

Algorithms used

1.1 Stepwise logistic regression based on P values with no adjustment (stepP)

The predictors were fitted into a multivariable, stepwise bidirectional selection procedure based on [1]. Starting from a model with all predictors included, a stepwise selection procedure was used to remove variables based on P >0.05. As some removed variables might improve the model once other predictors are removed, the procedure also allows adding back already removed variables. No adjustment was made to the p-value threshold.

1.2 Stepwise logistic regression based on P values with adjustment (stepPAdj)

The same multivariable, stepwise bidirectional selection procedure based on [1] was used as above, but the p-value was adjusted using the Bonferroni method [2].

1.3 Stepwise logistic regression based on AIC (stepAIC)

Similar to the stepwise selection using p-values, a logistic regression with all predictors included was fitted, followed by a bidirectional stepwise selection procedure that either removes one variable from the model or adds one variable again to the model. This is done by comparing all models with the AIC and choosing the one with the smallest AIC [3].

1.4 Best subset regression (BestSubset)

Best-subset selection aims to find a small subset of predictors such whilst retaining high prediction accuracy [4]. Using a 10-fold cross-validation (CV), an optimal number of predictors of the model is determined. Given this fixed model size, the model with the highest likelihood is found via a sequencing and splicing algorithm [4]. The resulting model is equal to penalizing the log-likelihood with a penalty that counts the number of non-zero coefficients.

1.5 Lasso

The LASSO regression constitutes a penalized linear model that aims to create the best-performing parsimonious model [5, 6]. It does so by adding a penalty equal to the absolute value of the magnitude of coefficients. Larger penalties result in coefficient values closer to zero, and some coefficients can become zero and be removed from the model. The LASSO produces coefficients that

are biased towards zero [7]. Hence, the predictors selected by LASSO were refitted with a simple logistic regression model to retrieve the unbiased coefficients.

1.6 Minimax concave penalty (MCP)

Similar to the Lasso and best subset selection, MCP is a penalized linear model that penalizes non-zero coefficients. For small coefficient magnitudes of regression coefficients, MCP's penalty is almost equivalent to the one from Lasso. For larger non-zero coefficient values, it relaxes the strength of regularization. In contrast to the Lasso, this allows for obtaining almost unbiased regression coefficients [7, 8].

1.7 Model-based boosting (mboost)

Model-based boosting uses a component-wise gradient boosting algorithm for model fitting [9]. The algorithm adds a predictor iteratively to the model to "correct" the error made by the prior model. The errors are defined by the first functional derivative of the current model and are regressed on every possible predictor using least squares. Subsequently, the best-fitting predictor is added to the model or its effect is updated if the predictor is already in the model. Given its iterative nature, some predictors are never selected, meaning that this method automatically performs predictor selection. To estimate the optimal number of iterations, a 10-fold CV was performed. The optimal iteration number is subsequently used to build and validate the final boosting model. The mboost produces coefficients that are biased towards zero [9]. Hence, the predictors selected by mboost were refitted with a simple logistic regression model to retrieve the unbiased coefficients.

1.8 MuARS

Multivariate adaptive regression splines are semi-parametric extensions of linear models to capture non-linear or interaction effects of predictors [10]. To allow for a fair comparison with other linear methods, no interactions were considered in this algorithm, although possible. The model is then built in an iterative manner considering all predictors that have already been added to the model in combination with all existing features. Similar to model-based boosting, the covariate is then greedily added to the model using least squares in a forward-pass and then the model is reduced in a backward step to avoid overfitting.

Prediction performance

Table S1. Prediction performance results of all models

Outcome	Model	Accuracy	AUC	Precision	Sensitivity	Specificity
Neck pain	Stepwise regression unadjusted P-values	0.763	0.618	0.557	0.322	0.913
Neck pain	Stepwise regression adjusted P-values	0.763	0.620	0.556	0.329	0.911
Neck pain	Stepwise regression AIC	0.760	0.613	0.545	0.316	0.911
Neck pain	Best subset regression	0.753	0.602	0.523	0.296	0.908
Neck pain	Lasso regression	0.753	0.607	0.522	0.309	0.904
Neck pain	Minimax concave penalty regression	0.753	0.602	0.523	0.296	0.908
Neck pain	Model based boosting	0.756	0.611	0.533	0.316	0.906
Neck pain	Multivariate adaptive regression splines	0.756	0.613	0.533	0.322	0.904
Arm pain	Stepwise regression unadjusted P-values	0.735	0.676	0.756	0.438	0.914
Arm pain	Stepwise regression adjusted P-values	0.733	0.676	0.746	0.442	0.909
Arm pain	Stepwise regression AIC	0.736	0.687	0.724	0.487	0.887
Arm pain	Best subset regression	0.738	0.688	0.728	0.487	0.890
Arm pain	Lasso regression	0.746	0.695	0.753	0.487	0.903
Arm pain	Minimax concave penalty regression	0.743	0.694	0.737	0.496	0.893
Arm pain	Model based boosting	0.746	0.694	0.757	0.482	0.906
Arm pain	Multivariate adaptive regression splines	0.738	0.688	0.728	0.487	0.890
Disability	Stepwise regression unadjusted P-values	0.618	0.619	0.676	0.464	0.774
Disability	Stepwise regression adjusted P-values	0.554	0.556	0.609	0.325	0.788
Disability	Stepwise regression AIC	0.634	0.635	0.667	0.550	0.721
Disability	Best subset regression	0.641	0.642	0.676	0.553	0.731
Disability	Lasso regression	0.634	0.635	0.669	0.543	0.727
Disability	Minimax concave penalty regression	0.636	0.637	0.669	0.550	0.724
Disability	Model based boosting	0.629	0.630	0.668	0.526	0.734
Disability	Multivariate adaptive regression splines	0.631	0.632	0.678	0.510	0.754

Coefficient strength heatmap

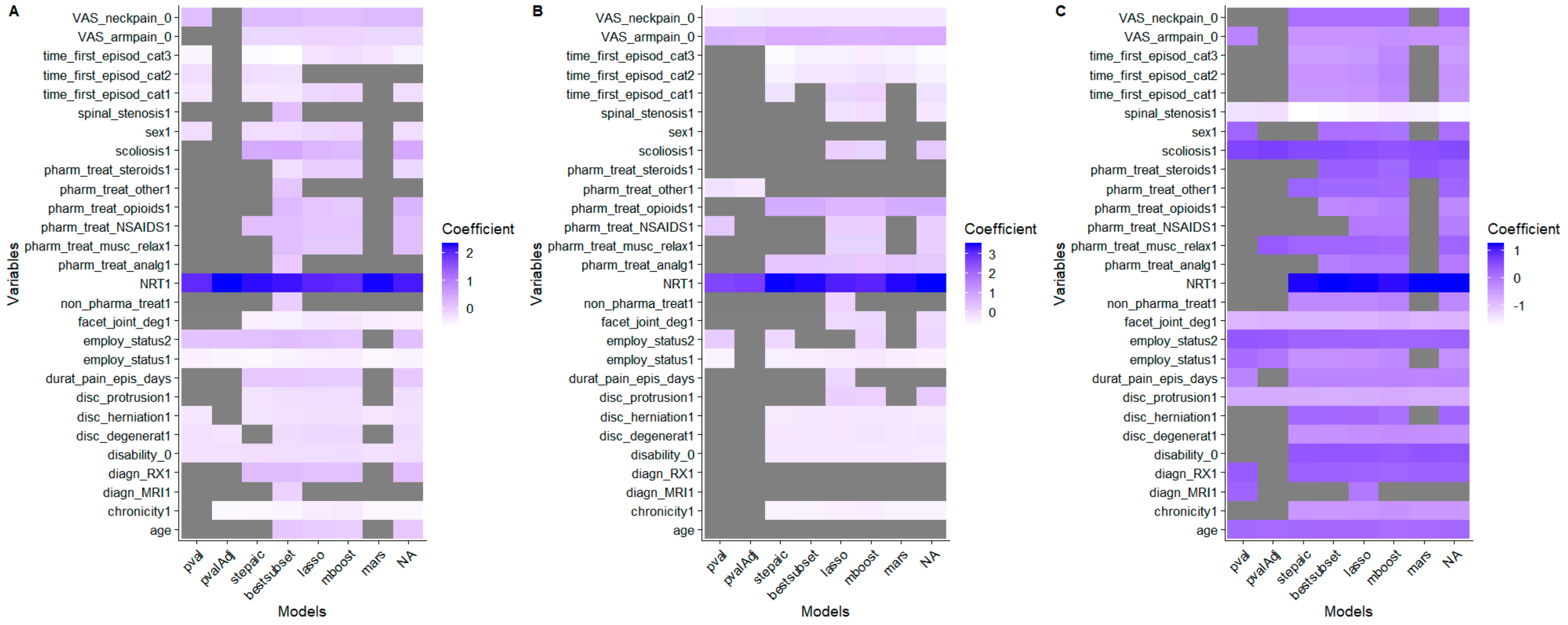

Figure S1. Results of variable selection for the outcomes of (a) neck pain, (b) arm pain, and (c) disability. Tiles shaded grey represent variables not selected. Abbreviations. stepP: Stepwise logistic regression based on P values with no adjustment; stepPAdj: Stepwise logistic regression based on P values with adjustment; stepAIC: Stepwise logistic re-gression based on AIC; BestSubset: Best subset regression; Lasso: least absolute shrinkage and selection operator; MuARS: multivariate adaptive regression spline; MCP: Minimax concave penalty; mboost: Model-based boosting; area under the receiver operating characteristic curve (AUC).

Receiver operating characteristic curves

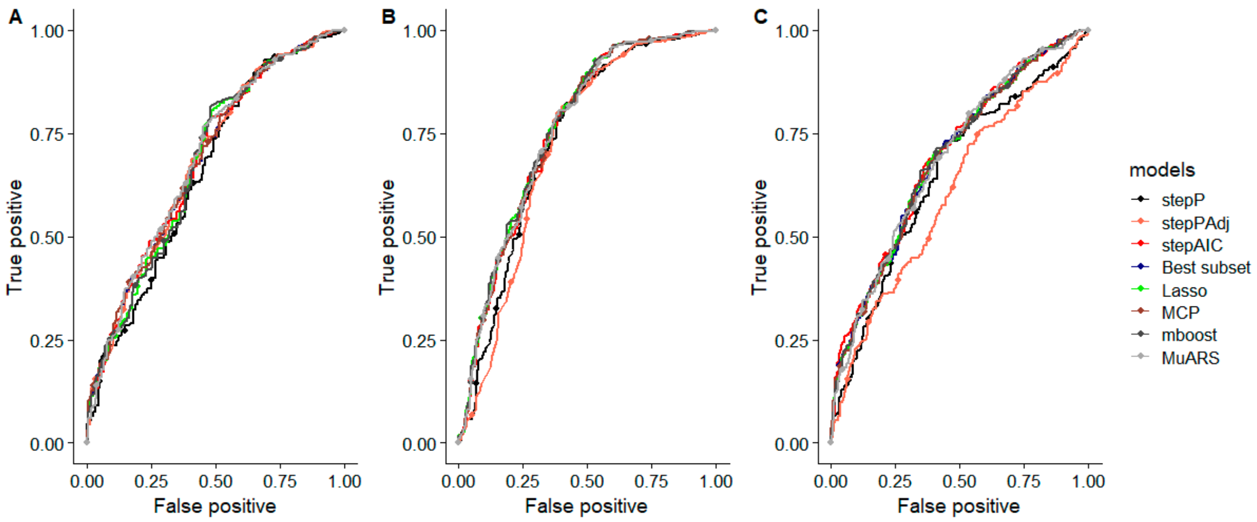

Figure S2. Receiver operating characteristic curves for all machine learning algorithms for the outcomes of (a) neck pain, (b) arm pain, and (c) disability. **Abbreviations.** stepP: Stepwise logistic regression based on P values with no adjustment; stepPAdj: Stepwise logistic regression based on P values with adjustment; stepAIC: Stepwise logistic re-gression based on AIC; BestSubset: Best subset regression; Lasso: least absolute shrinkage and selection operator; MuARS: multivariate adaptive regression spline; MCP: Minimax concave penalty; mboost: Model-based boosting; area under the receiver operating characteristic curve (AUC).

References

1. Zambom AZ, Kim J. Consistent significance controlled variable selection in high-dimensional regression. Stat. 2018;7(1):e210.

2. Yoav B, Daniel Y. The control of the false discovery rate in multiple testing under dependency. Ann Stat. 2001;29(4):1165-88.

3. Akaike H. A new look at the statistical model identification. IEEE Transactions on Automatic Control. 1974;19(6):716-23.

4. Zhu J, Hu L, Huang J, Jiang K, Zhang Y, Lin S, et al. abess: A Fast Best Subset Selection Library in Python and R. arXiv preprint arXiv:211009697. 2021.

5. Tibshirani R. Regression Shrinkage and Selection via the Lasso. Journal of the Royal Statistical Society Series B (Methodological). 1996;58(1):267-88.

6. Moons KGM, Altman DG, Reitsma JB, Ioannidis JPA, Macaskill P, Steyerberg EW, et al. Transparent Reporting of a multivariable prediction model for Individual Prognosis Or Diagnosis (TRIPOD): Explanation and Elaboration. Ann Intern Med. 2015;162(1):W1-W73.

7. Cun-Hui Z. Nearly unbiased variable selection under minimax concave penalty. Ann Stat. 2010;38(2):894-942.

8. Breheny P, Huang J. Coordinate descent algorithms for nonconvex penalized regression, with applications to biological feature selection Ann Appl Stat. 2011;5(1):232-53.

9. Buhlmann P, Hothorn T. Boosting Algorithms: Regularization, Prediction and Model Fitting. Statist Sci. 2007;22(4):477-505.

10. Friedman JH. Multivariate Adaptive Regression Splines. Ann Statist. 1991;19(1):1-67.
